# Collaboration Structures in COVID-19 Critical Care: Retrospective Network Analysis Study

**DOI:** 10.2196/25724

**Published:** 2021-03-08

**Authors:** Chao Yan, Xinmeng Zhang, Cheng Gao, Erin Wilfong, Jonathan Casey, Daniel France, Yang Gong, Mayur Patel, Bradley Malin, You Chen

**Affiliations:** 1 Department of Electrical Engineering & Computer Science Vanderbilt University Nashville, TN United States; 2 Department of Biomedical Informatics Vanderbilt University Medical Center Nashville, TN United States; 3 Division of Allergy, Pulmonary and Critical Care Medicine Vanderbilt University Medical Center Nashville, TN United States; 4 Department of Anesthesiology Vanderbilt University Medical Center Nashville, TN United States; 5 School of Biomedical Informatics University of Texas Health Science Center at Houston Houston, TX United States; 6 Department of Hearing & Speech Sciences Vanderbilt University Medical Center Nashville, TN United States; 7 Geriatric Research and Education Clinical Center Veteran Affairs Tennessee Valley Healthcare System Nashville, TN United States; 8 Department of Surgery Vanderbilt University Medical Center Nashville, TN United States; 9 Department of Neurosurgery Vanderbilt University Medical Center Nashville, TN United States; 10 Department of Biostatistics Vanderbilt University Medical Center Nashville, TN United States

**Keywords:** COVID-19, intensive care unit, collaboration structure, critically ill patient, health care worker, network analysis, electronic health record, collaboration, critical care, relationship, safety, teamwork

## Abstract

**Background:**

Few intensive care unit (ICU) staffing studies have examined the collaboration structures of health care workers (HCWs). Knowledge about how HCWs are connected to the care of critically ill patients with COVID-19 is important for characterizing the relationships among team structures, care quality, and patient safety.

**Objective:**

We aimed to discover differences in the teamwork structures of COVID-19 critical care by comparing HCW collaborations in the management of critically ill patients with and without COVID-19.

**Methods:**

In this retrospective study, we used network analysis methods to analyze the electronic health records (EHRs) of 76 critically ill patients (with COVID-19: n=38; without COVID-19: n=38) who were admitted to a large academic medical center, and to learn about HCW collaboration. We used the EHRs of adult patients who were admitted to the COVID-19 ICU at the Vanderbilt University Medical Center (Nashville, Tennessee, United States) between March 17, 2020, and May 31, 2020. We matched each patient according to age, gender, and their length of stay. Patients without COVID-19 were admitted to the medical ICU between December 1, 2019, and February 29, 2020. We used two sociometrics—eigencentrality and betweenness—to quantify HCWs’ statuses in networks. Eigencentrality characterizes the degree to which an HCW is a core person in collaboration structures. Betweenness centrality refers to whether an HCW lies on the path of other HCWs who are not directly connected. This sociometric was used to characterize HCWs’ broad skill sets. We measured patient staffing intensity in terms of the number of HCWs who interacted with patients’ EHRs. We assessed the statistical differences in the core and betweenness statuses of HCWs and the patient staffing intensities of COVID-19 and non–COVID-19 critical care, by using Mann-Whitney U tests and reporting 95% CIs.

**Results:**

HCWs in COVID-19 critical care were more likely to frequently work with each other (eigencentrality: median 0.096) than those in non–COVID-19 critical care (eigencentrality: median 0.057; *P*<.001). Internal medicine physicians in COVID-19 critical care had higher core statuses than those in non–COVID-19 critical care (*P*=.001). Nurse practitioners in COVID-19 care had higher betweenness statuses than those in non–COVID-19 care (*P*<.001). Compared to HCWs in non–COVID-19 settings, the EHRs of critically ill patients with COVID-19 were used by a larger number of internal medicine nurse practitioners (*P*<.001), cardiovascular nurses (*P*<.001), and surgical ICU nurses (*P*=.002) and a smaller number of resident physicians (*P*<.001).

**Conclusions:**

Network analysis methodologies and data on EHR use provide a novel method for learning about differences in collaboration structures between COVID-19 and non–COVID-19 critical care. Health care organizations can use this information to learn about the novel changes that the COVID-19 pandemic has imposed on collaboration structures in urgent care.

## Introduction

The COVID-19–Associated Hospitalization Surveillance Network has reported that the overall cumulative COVID-19 hospitalization rate in the United States is 199.8 people per 100,000 people for the week that ended October 24, 2020 [1]. Additionally, between 5% and 12.2% of patients aged <60 years and between 27.4% and 70.9% of patients aged ≥60 years have required intensive care due to deteriorating respiratory conditions [2-4]. Health care organizations (HCOs) have been exploring various approaches (eg, the creation of COVID-19 intensive care units [ICUs] and the extension of existing ICUs) for satisfying the increasing medical needs of critically ill patients with COVID-19 [5-7]. Various staffing strategies and protocols for the care of critically ill patients with COVID-19 have been developed [8-10]. These strategies belong to three ICU staffing model categories—open, closed, and hybrid. In an open model, many different medical staff members manage patients in ICUs. In contrast, the closed model limits the staffing system to ICU-certified physicians (eg, intensivists). The hybrid model draws upon the aspects of the open and closed models by staffing ICUs with an attending physician and a team so that they can work in tandem with primary physicians.

ICU staffing (eg, the assignment of patients to a set of health care workers [HCWs]) can impact care quality and patient safety [11-13]. As such, HCOs need to be mindful of how they assess collaborations among HCWs to properly care for critically ill patients with COVID-19. However, COVID-19 ICU staffing strategies are designed at a very high level (eg, team scheduling). Therefore, they neglect the cross-disciplinary connections among HCWs. Gaining knowledge on how HCWs connect and collaborate can improve teamwork, which in turn may improve care quality and patient safety [14].

Few studies have investigated the collaboration structures of COVID-19 critical care [15], but to the best of our knowledge, none have examined the collaborations among HCWs. As such, there is a limited amount of explicitly documented evidence about cross-disciplinary (eg, internal medicine physicians, respiratory therapists, and cardiovascular nurses) collaboration in COVID-19 critical care. HCOs need this information to manage teamwork and improve care quality and patient safety during the pandemic. In this study, we used network analysis methods to learn about the collaboration structures of COVID-19 critical care. We specifically investigated how HCWs are connected in the context of providing care to critically ill patients with COVID-19. One of the challenges in modeling the connections among HCWs in the ICU is their complexity (eg, cooperation among multidisciplinary HCWs). In this study, we learned about the collaborations among multidisciplinary HCWs by analyzing electronic health record (EHR) systems. EHR systems provide an environment that aids with teamwork (eg, the exchange of health information among HCWs). This can help HCOs with offering more accurate, detailed, and timely information, which would result in the delivery of higher quality care [16,17]. As EHR adoption has spread, the proportion of HCW activities (eg, the review of notes, requests for x-rays, and the management of medication) that involve EHRs has increased [18,19]. Thus, interactions with EHRs provide an opportunity for studying the collaborations among HCWs [20-24].

We conducted a secondary analysis on EHR use to learn about the collaborations among HCWs. We created networks by identifying connections among HCWs who conducted activities with the EHRs of the same patients on the same day.

We used two sociometrics—eigencentrality and betweenness centrality—to measure the core and betweenness status of an HCW in the collaboration network. Eigencentrality characterizes the degree to which an HCW is a core person in collaboration structures. Betweenness centrality refers to whether an HCW lies on the path of other HCWs who are not directly connected. An HCW who has a broad skill set and cares for a wide spectrum of patients could frequently be in a high-betweenness position.

We analyzed data on EHR system use in the Vanderbilt University Medical Center, which is a large academic medical center in Nashville, Tennessee that created its COVID-19 unit in the middle of March 2020. The high density of clinical ICU data and large volume of EHR activities for each ICU patient episode allow for the investigation of HCW collaboration in the management of critically ill patients before and during the COVID-19 pandemic.

We learned about the collaboration structures in COVID-19 critical care by comparing structures that were associated with the management of critically ill patients with and without COVID-19.

## Methods

### Data Set

We screened for adult patients who were admitted to the COVID-19 ICU between March 17, 2020, and May 31, 2020. We matched each patient with COVID-19 with an adult patient without COVID-19 who was admitted to the medical ICU (MICU) between December 1, 2019, and February 29, 2020, via propensity score matching.

The propensity score was based on age, gender, and patients’ length of stay. The distribution of the COVID-19 and non–COVID-19 groups’ propensity scores is depicted in Multimedia Appendix 1. The Pearson correlation coefficient between the two distributions was 0.93; the associated *P* value was <.001. This proved that the variance in the confounding factors between the two patient groups was very small. We focused on patients who were alive at discharge because their hospital stays were relatively complete. This process yielded a sample of 76 critically ill, adult patients—38 with COVID-19 and 38 without COVID-19. In total, 3 patients with COVID-19 required multiple ICU stays. For this study, we randomly selected one stay for each of these patients. Table 1 provides a summary of the demographic characteristics, comorbidities, and outcomes of the investigated patients with and without COVID-19.

**Table 1:** Characteristics of the critically ill patients in this study.

Characteristics			Patients with COVID-19^a^	Patients without COVID-19^b^
Patients, n			38	38
**Demographic characteristics**				
	Age (years), median (IQR; SD)		54 (47-66; 14)	54 (49-64; 12)
	**Sex, n (%)**			
		Female	15 (39)	15 (39)
		Male	23 (61)	23 (61)
	**Race, n (%)**			
		White	22 (58)	32 (84)
		African American	6 (16)	5 (13)
		Asian	4 (11)	0 (0)
		Other	6 (16)	1 (3)
**Outcomes**				
	Length of stay (days), median (IQR; SD)		13.5 (6.50-18.75; 10)	13.5 (7.50-19.00; 9)
	**Hospital discharge disposition, n (%)**			
		Home	29 (76)	24 (63)
		Other	9 (24)	14 (37)
**Comorbidities, n (%)**				
	Hypertension		19 (50)	33 (87)
	Cardiovascular disease		14 (37)	23 (61)
	Renal disease		19 (50)	22 (58)
	Diabetes		10 (26)	16 (42)
	Chronic metabolic disease		14 (37)	18 (47)
	Chronic lung disease		9 (24)	17 (45)

^a^Patients with COVID-19 were admitted to the intensive care unit between March 17, 2020, and May 31, 2020.

^b^Patients without COVID-19 were admitted to the medical intensive care unit between December 1, 2019, and February 29, 2020.

The study population had several notable aspects. First, we noticed that there was a disproportionate number of males. Second, while there were more self-reported White patients than patients of other races, the number of White patients in the COVID-19 group was substantially smaller than that in the non–COVID-19 group. Third, patients without COVID-19 had a high incidence of comorbidities; specifically, patients without COVID-19 exhibited the six comorbidities that are common in patients with COVID-19 (ie, those reported by the COVID-19–Associated Hospitalization Surveillance Network) [1]. Fourth, the majority of patients from the two groups (with COVID-19: 29/38, 76%; without COVID-19: 24/38, 63%) were discharged home.

### Study Design

The analysis consisted of two primary components. First, we used network analysis methods to learn about the HCW networks that were involved in the management of critically ill patients. Second, we statistically compared and contrasted the network structures in COVID-19 and non–COVID-19 settings.

### Modeling HCW Networks

We analyzed the actions that HCWs performed with patients’ EHRs to measure worker-worker connections. There are six types of HCW actions, including condition-related (eg, assigning a diagnosis), procedure-related (eg, intubation), medication-related (eg, prescriptions), note-related (eg, writing progress notes), order-related (eg, ordering laboratory tests), and measurement-related (eg, measuring respiratory rate) actions.

Research has shown that a 1-day window is enough to capture the meaningful, collaborative relationships among HCWs [20-23]. Therefore, we assumed that there was a connection between two HCWs who interacted with the same patient’s EHR on the same day. We built a network in which the nodes represented HCWs and the edges indicated the number of days that two HCWs performed actions on the EHRs of the same patients. We built one network for critically ill patients with COVID-19 and another for patients without COVID-19.

The nodes in the COVID-19 and non–COVID-19 networks were defined as follows:

Z_COVID-19_ = {z_1_, z_2_,…, z_p_} **(1)**

Z_Non–COVID-19_ = {z'_1_, z'_2_,…, z'_q_} **(2)**

To better interpret the networks, we used an HCW’s specialty (eg, respiratory care) and type (eg, respiratory therapist) to label each node. We combined these factors to define expertise (ie, “specialty: type”; eg, respiratory care: respiratory therapist). Expertise in the COVID-19 and non–COVID-19 networks were defined as follows:

EXP_COVID-19_ = {exp_1_, exp_2_,…, exp_a_} **(3)**

EXP_Non–COVID-19_ = {exp'_1_, exp'_2_,…, exp'_b_} **(4)**

In equations 1-4, Z and EXP were used to describe the composition of COVID-19 or non–COVID-19 networks.

In each network, we used two sociometrics—eigenvector centrality and betweenness centrality—to quantify an HCW’s core and betweenness status in the network, respectively. We used Gephi (ie, an open-source network analysis and visualization software package) [25] to calculate eigencentrality and betweenness centrality values.

Eigencentrality characterizes the degree to which an HCW is densely connected to other HCWs who are also densely connected with other HCWs. A high-eigencentrality HCW is likely to be a core person who actively works with other HCWs when performing actions on EHRs. An example HCW network with eigencentrality values is shown in Multimedia Appendix 2.

The betweenness centrality of an HCW refers to the number of shortest paths between two other HCWs that pass through the HCW in question. An HCW with a broad skill set who cares for a wide spectrum of patients could frequently be in a high-betweenness position. An example HCW network with betweenness centrality values is shown in Multimedia Appendix 2.

### Eigencentrality and Betweenness in COVID-19 and Non–COVID-19 Networks

We investigated whether differences in the eigencentrality and betweenness of COVID-19 and non–COVID-19 critical care structures were statistically significant at the network and expertise levels. The network-level comparison was conducted by assessing the network as a whole (ie, COVID-19 vs non–COVID-19 networks), while an expertise-level comparison was conducted for each expertise (eg, internal medicine physicians). Since eigencentrality and betweenness are not Gaussian distributed, we conducted a Mann-Whitney U test with a significance level of α=.05. The tests for expertise included at least 8 HCWs and involved Bonferroni correction to account for multiple hypotheses.

### Patient Staffing Intensity in COVID-19 and Non–COVID-19 Settings

We defined the set of inpatient stays in COVID-19 and non–COVID-19 settings as follows:

S_COVID-19_ = {s_1_, s_2_,…, s_m_} **(5)**

S_Non–COVID-19_ = {s'_1_, s'_2_,…, s'_n_} **(6)**

Since each inpatient stay (ie, s_i_) can last for more than 1 day, we defined the jth day of a stay as s_i,j_ (ie, 1≤j≤l_i_); l_i_ represents the last day of a patient’s hospital stay (ie, s_i_). For s_i,j_, we calculated the number of HCWs (ie, Ns_i,j,_exp_k_) in each expertise category (ie, exp_k_) who interacted with the EHRs of patient i on day j. For each inpatient stay (ie, s_i_), we calculated the average number of HCWs in each expertise category (ie, exp_k_) who interacted with the EHRs of the same patient on each day, as follows:


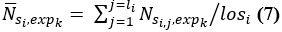


In equation 7, los_i_ refers to the length of hospital stay (ie, the total number of hours between the start and end times of an inpatient stay divided by 24 hours). Since each inpatient stay may start and end at different times of the day,los_i_ may be different from l_i_. Daily patient staffing intensity was defined as an expertise-level value (ie, 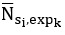).

To learn about the differences in the daily patient staffing intensities of COVID-19 and non–COVID-19 critical care, we conducted a set of tests. Specifically, for each investigated expertise (eg, internal medicine nurse practitioners), we tested whether critically ill patients with COVID-19 required a significantly higher daily patient staffing intensity than critically ill patients without COVID-19. We focused on the 20 expertise categories with the highest mean daily staffing intensity values in COVID-19 and non–COVID-19 critical care and used the Mann-Whitney U test, which had a Bonferroni-corrected significance level of .05.

We also assessed the differences in the overall patient staffing intensities of COVID-19 and non–COVID-19 critical care in terms of the number of HCWs who were involved in the management of a patient. Overall staffing intensity was defined as the number of HCWs who interacted with the EHRs of a patient with or without COVID-19. Our hypothesis was as follows: critically ill patients with COVID-19 require a significantly higher overall staffing intensity than critically ill patients without COVID-19. We tested this hypothesis by using the Mann-Whitney U test, which had a Bonferroni-corrected significance level of .05.

## Results

### HCW Characteristics

The number of HCWs, types of HCWs, specialties, and expertise categories in COVID-19 and non–COVID-19 critical care was 759 and 1331, 24 and 24, 92 and 128, and 133 and 207, respectively. These values indicated that patients without COVID-19 required more expertise, specialties, and HCWs. A possible reason for this is that critically ill patients without COVID-19 were admitted to the MICU for a wide range of major conditions. With regard to patient-level values, COVID-19 and non–COVID-19 critical care consisted of 79.5 and 88.2 HCWs, 9.8 and 10.6 types of HCWs, 23.0 and 27.1 departments, and 29.2 and 34.0 expertise categories, respectively. The patient-level values for COVID-19 and non–COVID-19 critical care were highly similar.

**Figure 1:**
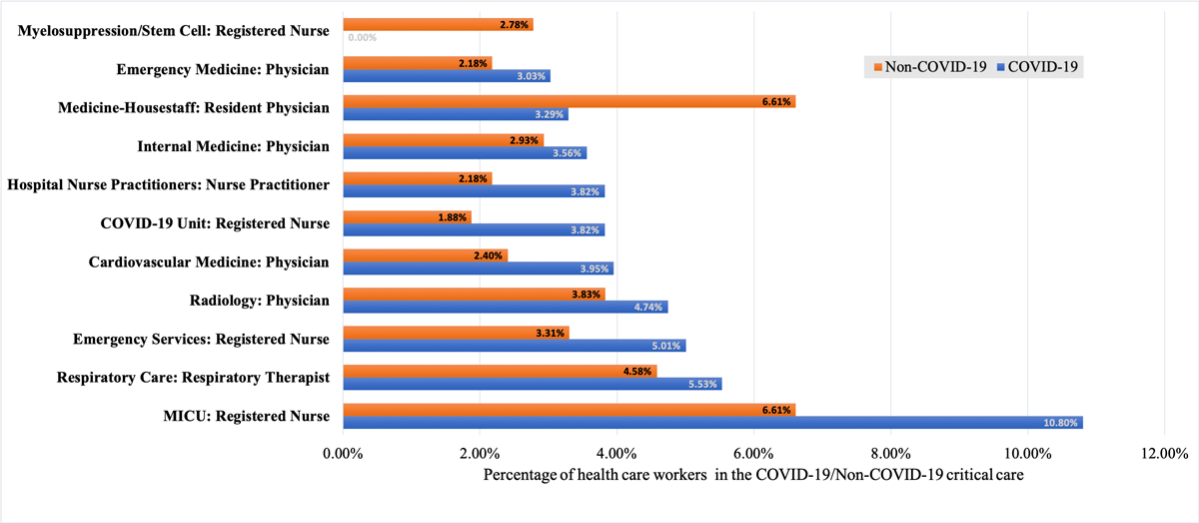
The expertise categories with the largest number of health care workers in the COVID-19 and non–COVID-19 settings. There are 11 expertise categories shown, which correspond to the union of the top 10 expertise categories in COVID-19 (ie, excluding nurses with myelosuppression expertise) and non–COVID-19 (ie, excluding COVID-19 unit nurses) critical care. Each expertise is reported in the following format: “specialty: health care worker type.” MICU: medical intensive care unit.

Figure 1 illustrates the union of the 10 COVID-19 and non–COVID-19 critical care expertise categories with the largest proportion of HCWs. It can be seen that, aside from residents and registered nurses with myelosuppression expertise, the COVID-19 setting had higher percentages of different types of HCWs than the non–COVID-19 setting. These results demonstrate how the Vanderbilt University Medical Center assigned full-time, nontrainee HCWs to the task of managing critically ill patients with COVID-19 and reduced the number of residents during the COVID-19 pandemic.

There were no registered nurses with myelosuppression expertise in the COVID-19 setting. Upon further analysis, we found 4 patients with COVID-19 and cancer, but none were in need of invasive intervention at the time of their care.

### Eigencentrality and Betweenness

**Figure 2:**
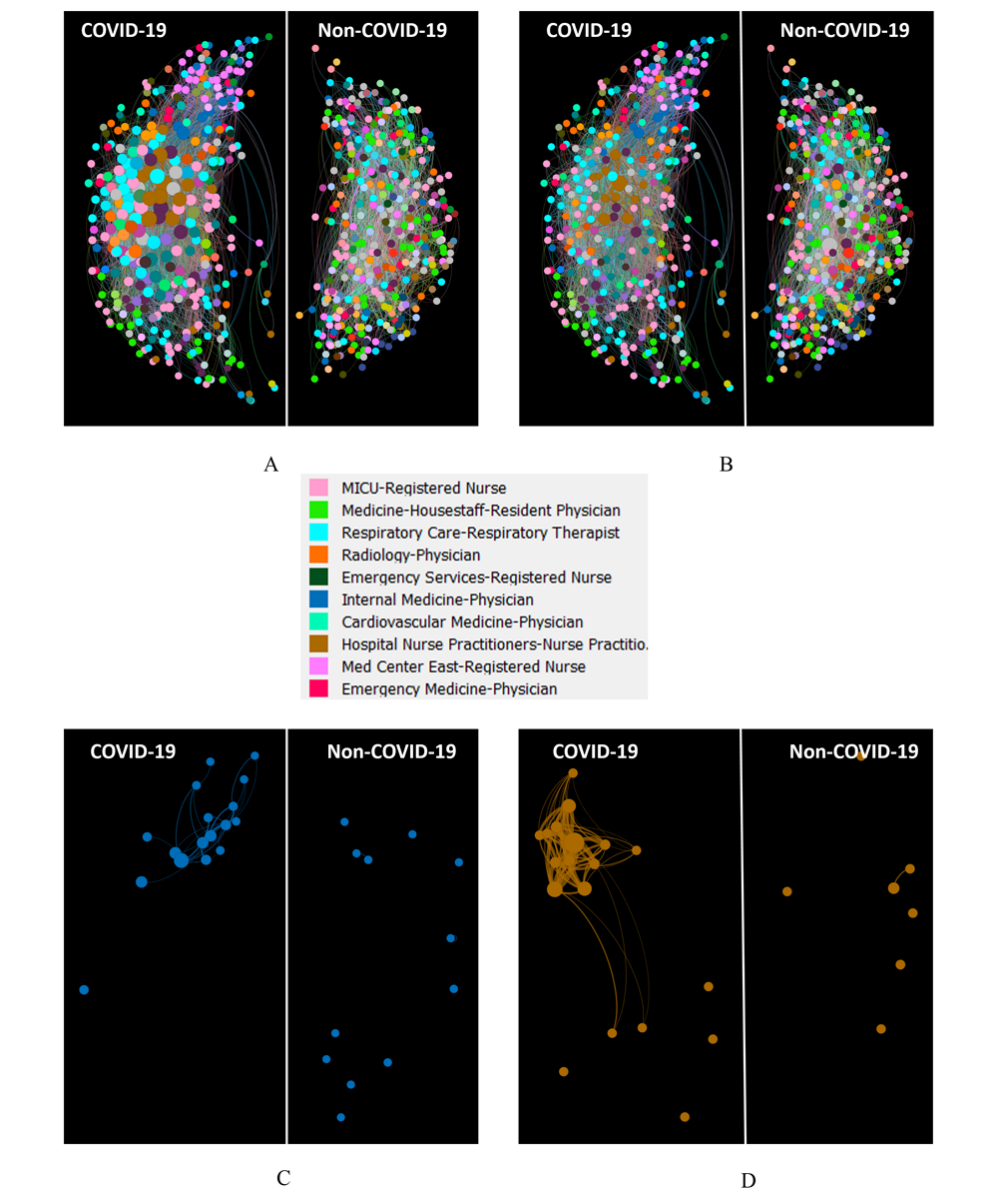
A depiction of the health care worker eigencentrality (A) and betweenness (B) in COVID-19 and non–COVID-19 networks. C and D show the subnetworks of internal medicine physicians and nurse practitioners in the COVID-19 and non–COVID-19 networks, respectively. In A and C, eigencentrality directly correlated with the size of the corresponding node. In B and D, betweenness centrality directly correlated with the size of the corresponding node. The legend in the figure shows the 10 expertise categories with the largest number of health care workers in the combined network (ie, both the COVID-19 and non–COVID-19 networks). MICU: medical intensive care unit.

Figure 2 presents the HCW networks in COVID-19 and non–COVID-19 critical care from eigencentrality and betweenness perspectives. In the figure, it can be seen that the majority of HCWs in the COVID-19 network are larger in size (ie, higher eigencentrality) than those in the non–COVID-19 network. This indicates that HCWs are much more highly and densely connected in the COVID-19 network than those in the non–COVID-19 network. We performed a test to measure the differences in eigencentrality between the COVID-19 and non–COVID-19 networks. The results indicated that the two networks had significantly different median eigencentrality values (COVID-19: 0.096; non–COVID-19: 0.057; *P<.*001).

**Figure 3:**
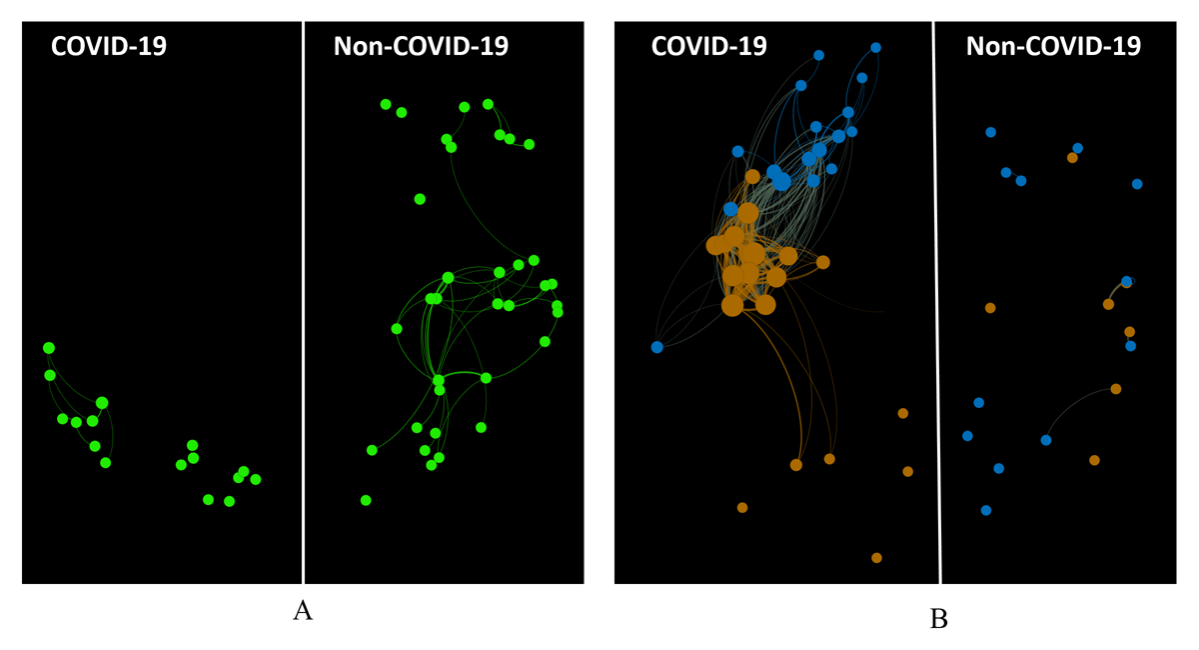
(A) The subnetworks of resident physicians in the COVID-19 and non–COVID-19 networks. (B) The subnetworks of internal medicine physicians and nurse practitioners in the COVID-19 and non–COVID-19 networks. Eigencentrality directly correlated with the size of the corresponding node in both A and B.

After removing expertise categories that had less than 8 HCWs, we performed pairwise tests on the remaining 12 expertise categories. The results of these tests are provided in Table S1 in Multimedia Appendix 3. There were several notable findings. First, we observed that internal medicine physicians in the COVID-19 network had higher eigencentrality values than those in the non–COVID-19 network (*P*=.001). Figure 2 also shows the subnetworks of internal medicine physicians in the COVID-19 and non–COVID-19 networks from an eigencentrality perspective. From the figure, it can be seen that internal medicine physicians in the COVID-19 network were connected with each other, while those in the non–COVID-19 network were separated. Second, the resident physicians in the non–COVID-19 network had higher eigencentrality values than those in the COVID-19 network (*P*=.002). Figure 3 shows that there were many residents across the entire non–COVID-19 network. However, the number of residents was much smaller in the COVID-19 network.

There were no significant differences in the betweenness of the two networks (COVID-19 network: median 0.002; non–COVID-19 network: median 0.003; *P*=.22). However, nurse practitioners in the COVID-19 network had significantly higher betweenness values than those in the non–COVID-19 network (*P*<.001). The complete set of test results for expertise categories with nonsignificantly different betweenness values is provided in Table S2 in Multimedia Appendix 3. Figure 2 also shows the subnetworks of nurse practitioners in the COVID-19 and non–COVID-19 networks from a betweenness perspective. From the figure, it can be seen that nurse practitioners had a larger number of connections in the COVID-19 network than those in the non–COVID-19 network. In Figure 2, it can also be seen that nurse practitioners are in the central part of the COVID-19 network and serve as connective bridges between other HCWs. Given that betweenness reflects an HCW’s access to a wide spectrum of patients, a nurse practitioner in the COVID-19 collaboration structure can build connections among HCWs who are not directly connected. Internal medicine physicians and nurse practitioners were the core of the COVID-19 network. As shown in Figure 3, these two types of HCWs had a larger number of connections in the COVID-19 care setting than those in the non–COVID-19 setting.

### Differences in Patient Staffing Intensity

In total, 41,903 (mean 1103) actions were performed with the EHRs of patients with COVID-19 and 44,131 (mean 1161) actions were performed with the EHRs of patients without COVID-19. There were no statistically significant differences in the number of actions performed with the EHRs of patients with and without COVID-19 (*P*=.32). The differences in the number of expertise categories and HCWs who performed actions on the EHRs of critically ill patients with and without COVID-19 were also not significant (expertise categories: *P*=.08; HCWs: *P*=.19). The complete set of results is provided in Table S3 in Multimedia Appendix 4.

Table 2 shows the differences in the patient staffing intensities of COVID-19 and non–COVID-19 critical care for each expertise category. The union of 20 COVID-19 and 20 non–COVID-19 expertise categories yielded 24 categories.

**Table 2:** Differences in the daily average patient staffing intensities of COVID-19 and non–COVID-19 critical care. A Bonferroni-corrected *P* value of .002 was used as the null hypothesis rejection threshold.

Expertise category		Staffing intensity, mean (SD)		Staffing intensity, median (IQR)		*P* value
		COVID-19 critical care	Non–COVID-19 critical care	COVID-19 critical care	Non–COVID-19 critical care	
**COVID-19 categories**						
	Hospital nurse practitioners: nurse practitioner	1.81 (0.19)	0.27 (0.10)	1.76 (1.37)	0 (0.18)	<.001
	Medical Center East^a^: registered Nurse	0.78 (0.08)	0.21 (0.07)	0.69 (0.70)	0 (0.02)	<.001
	Cardiovascular intensive care unit: registered Nurse	0.26 (0.05)	0.01 (0.01)	0.18 (0.37)	0 (0)	<.001
	Internal medicine: nurse practitioner	0.33 (0.05)	0.06 (0.02)	0.23 (0.30)	0 (0.05)	<.001
	Surgical intensive care unit: registered nurse	0.17 (0.04)	0.03 (0.02)	0.09 (0.29)	0 (0)	.002
**Non–COVID-19 categories**						
	Medicine house staff: resident physician	0.18 (0.06)	1.56 (0.15)	0 (0.07)	1.62 (1.32)	<.001
	Emergency medicine: resident physician	0.06 (0.03)	0.27 (0.05)	0 (0.05)	0.16 (0.30)	<.001
**Categories that were not statistically significant**						
	Medical Center East^a^: technician	0.23 (0.03)	0.10 (0.04)	0.22 (0.19)	0 (0)	.007
	Hematology oncology: physician	3.54 10^-3^ (2.87 10^-3^)	0.22 (0.08)	0 (0)	0 (0.06)	.01
	Emergency medicine: physician	0.27 (0.06)	0.53 (0.10)	0.11 (0.43)	0.30 (0.92)	.02
	Pharmacy inpatient (central): pharmacist	0.12 (0.03)	0.21 (0.03)	0.06 (0.14)	0.15 (0.13)	.03
	Pharmacy inpatient (evening): pharmacist	0.16 (0.03)	0.24 (0.03)	0.12 (0.28)	0.21 (0.23)	.03
	Infectious disease: physician	0.05 (0.03)	0.18 (0.05)	0 (0)	0 (0.20)	.05
	Internal medicine: physician	1.14 (0.13)	0.60 (0.11)	0.92 (1.12)	0.53 (0.90)	.002
	Medical intensive care unit: registered nurse	1.19 (0.13)	0.95 (0.10)	1.03 (0.91)	0.85 (0.78)	.13
	Radiology: physician	0.30 (0.04)	0.38 (0.04)	0.27 (0.34)	0.37 (0.42)	.15
	Nephrology: physician	0.14 (0.05)	0.26 (0.10)	0 (0)	0 (0.05)	.21
	Allergy/pulmonary: physician	0.80 (0.11)	0.94 (0.12)	0.65 (0.82)	0.88 (0.73)	.21
	Pharmacy inpatient operations manager: pharmacist	0.14 (0.02)	0.21 (0.04)	0.11 (0.13)	0.14 (0.24)	.22
	Pharmacy inpatient satellite operating room: pharmacist	0.20 (0.04)	0.15 (0.03)	0.11 (0.38)	0.11 (0.23)	.28
	Respiratory care: respiratory therapist	0.74 (0.11)	0.89 (0.14)	0.60 (0.87)	0.80 (1.32)	.30
	Emergency services: registered nurse	0.15 (0.05)	0.17 (0.05)	0.07 (0.18)	0.09 (0.18)	.41
	Pharmacy inpatient (evening): pharmacy technician	0.26 (0.05)	0.28 (0.05)	0.16 (0.36)	0.19 (0.40)	.43
	Cardiovascular medicine: physician	0.21 (0.04)	0.18 (0.03)	0.15 (0.37)	0.16 (0.15)	.43

^a^Medical Center East is the building where we created the COVID-19 unit. Before the creation of the COVID-19 unit, nurses in this building cared for critically ill patients without COVID-19.

There was a larger number of internal medicine nurse practitioners, cardiovascular ICU registered nurses, and surgical ICU registered nurses who performed daily actions on the EHRs of critically ill patients with COVID-19 compared to the number of those who performed daily actions on the EHRs of patients without COVID-19. In contrast, the EHRs of patients without COVID-19 were managed by a larger number of resident physicians (ie, those with medicine and emergency medicine expertise). These differences were statistically significant (Table 2).

We also found that expertise categories were not statistically different in terms of daily patient staffing intensity. Such categories included radiology physicians, nephrology physicians, pulmonary/allergy physicians, emergency medicine physicians, MICU registered nurses, and respiratory therapists.

## Discussion

### Principal Findings

There are no universal guidelines for HCW staffing in ICUs. To date, ICU staffing studies have focused on organization models (eg, open, closed, and hybrid models), and few have examined collaborations among HCWs. In this study, we used a novel method for learning about collaborations among HCWs and building corresponding networks. We measured eigencentrality and betweenness centrality to quantify the core and betweenness statuses of HCWs and identify several significant differences between the COVID-19 and non–COVID-19 network structures. Differences in the collaboration structures between the two networks mirrored those in intentional strategic planning structures across the health care system. For instance, there was a significant difference (*P*<.001) in the number of resident physicians between the COVID-19 and non–COVID-19 structures because our medical center assigned full-time, nontrainee HCWs to the management of critically ill patients with COVID-19. This mirrors resident protection strategies that were implemented during the outset of the COVID-19 pandemic by the National Graduate Medical Education. Figure 3 shows the subnetworks of resident physicians in the COVID-19 and non–COVID-19 networks. It can be seen that the non–COVID-19 network has a larger resident network than the COVID-19 network, and the connections between residents are more complex than those in the COVID-19 network. This suggests that resident physicians are highly active with respect to the management of critically ill patients in a non–COVID-19 setting.

Beyond collecting data on basic strategic planning methods (ie, the reduction of the number of residents) in the management of critically ill patients with COVID-19, we also learned about the aspects of collaboration structures that are important for the management of teamwork but are not explicitly documented in existing ICU staffing plans. We found that internal medicine physicians and nurse practitioners in the COVID-19 collaboration structure were more active (ie, high eigencentrality or betweenness) than those in the non–COVID-19 collaboration structure. As shown in Figure 3, internal medicine physicians and nurse practitioners connected more frequently in the COVID-19 network than those in the non–COVID-19 network. This phenomenon suggests that they are core members in collaborations that relate to the management of critically ill patients with COVID-19.

Combining knowledge on connections among HCWs with their eigencentrality and betweenness values in the collaboration network can assist HCOs with designing and developing more specific staffing strategies, which can potentially improve care quality and patient outcomes. The network analysis methods and team structures that are depicted in our retrospective study can be used in a prospective setting. Our COVID-19 and non–COVID-19 networks can be used to identify the characteristics of a newly established or modified team. For instance, if a COVID-19 ICU has plans for creating a team to care for the increasing number of patients, the eigencentrality and betweenness centrality of each HCW and the HCW relationships that we learned about in our COVID-19 network can be used as evidence for identifying the characteristics of the newly created team. The team creators can evaluate the leadership (ie, eigencentrality), robustness (ie, betweenness), and familiarity (ie, the strength of the relationships between HCWs) of the newly established team. They can also dynamically add or remove an HCW from the created team and measure changes in leadership, robustness, and familiarity, which will help team creators with finding their desired team.

### The Scope of This Study and Its Limitations

In this study, we did not investigate temporal networks or team dynamics, which are essential to HCOs that monitor and manage team dynamics. However, researchers can use the network analysis methodologies that were developed in our study to identify temporal networks, such as daily, weekly, or monthly networks. They can also use the sociometrics that we developed to quantify changes in temporal network structures. For instance, HCOs can use our network analysis methodologies to temporally measure the relationships among internal medicine physicians, nurse practitioners, and residents; and quantify the weekly, monthly, and yearly changes in these relationships.

There are several limitations in this study that should be recognized. These limitations serve as opportunities for further investigation. First, this study was based on a small number of critically ill patients with COVID-19. Although our sample had sufficient power for analyzing the differences in the eigencentrality, betweenness, and patient staffing intensities for several types of HCWs in COVID-19 and non–COVID-19 critical care, a larger volume of data is needed to obtain statistically meaningful results. Second, comorbidities could have impacted team structures; however, matching the comorbidities between patients with and without COVID-19 can lead to certain risks. According to our observations, matching comorbidities between the two cohorts will considerably enlarge the study window (ie, >3 years) for the non–COVID-19 cohort. However, non–COVID-19 care teams can drastically change over time, making the study of the non–COVID-19 team structures less meaningful. Therefore, in this study, we focused on the most important confounding factors (ie, age, gender, and the length of stay) that characterize team efficacy and may impact team structures. Additionally, our study's primary focus was to learn about team structures in COVID-19 and medical ICUs, which allowed for some degree of variance in comorbidities. Third, the characteristics of the COVID-19 structures that we learned about during this single-center study could provide HCOs with reference data for assessing their own COVID-19 ICU structures. However, our medical center is an institution that intentionally developed a nurse practitioner–centered organizational structure. This should be considered when interpreting our results and findings. To learn about general COVID-19 ICU collaboration structures, researchers need to conduct analyses that account for multiple HCOs. Fourth, there was a lack of standard terminology for characterizing HCO departments and the roles of HCWs. Although there are taxonomies for describing clinician specialties [26-28], these tend to neglect the nonphysicians who play vital roles in the management of patients. It is clear that common data models for department names and HCW types would improve the quality of our study and assist other institutions with using our methodology. Fifth, we assumed that two HCWs would have a connection when they performed actions on the EHRs of patients. Although such an assumption can help with identifying collaboration relationships between HCWs, it may also have resulted in the identification of many spurious relationships.

### Conclusion

HCOs have been planning and refining their staffing strategies to provide more efficient and effective care to patients with COVID-19. However, there are few efficient methodologies for assessing the execution of collaboration structures in practice, especially those for assessing the cross-disciplinary connections among HCWs. In this study, we demonstrated how data on the use of a large academic medical center’s EHR system could be used to learn about the collaboration structures in COVID-19 critical care (ie, through network analysis methodologies). HCOs can use our network analysis approaches and data on eigencentrality, betweenness, and patient staffing intensities to characterize HCW roles in collaboration networks during the COVID-19 pandemic or future events. Furthermore, research on how HCWs are connected has created an opportunity for studying the relationships among team structures, care quality, and patient safety.

## Appendix

Multimedia Appendix 1 Distributions of the COVID-19 and non–COVID-19 groups' propensity scores.

Multimedia Appendix 2 Examples that illustrate eigencentrality and betweenness centrality.

Multimedia Appendix 3 Differences in the eigencentrality and betweenness of COVID-19 and non–COVID-19 structures.

Multimedia Appendix 4 Differences in overall patient staffing intensities between the COVID-19 and non–COVID-19 structures.

## Abbreviations

EHR: electronic health record
HCO: health care organization
HCW: health care worker
ICU: intensive care unit
MICU: medical intensive care unit
