# Supplementary material for: Collaboration Structures in COVID-19 Critical Care: Retrospective Network Analysis Study
Source: JMIR Hum Factors. 2021 Mar 8;8(1):e25724. doi: 10.2196/25724 (PMC7942392; doi:10.2196/25724)
Supplement: Multimedia Appendix 1 [file humanfactors_v8i1e25724_app1.docx]

**
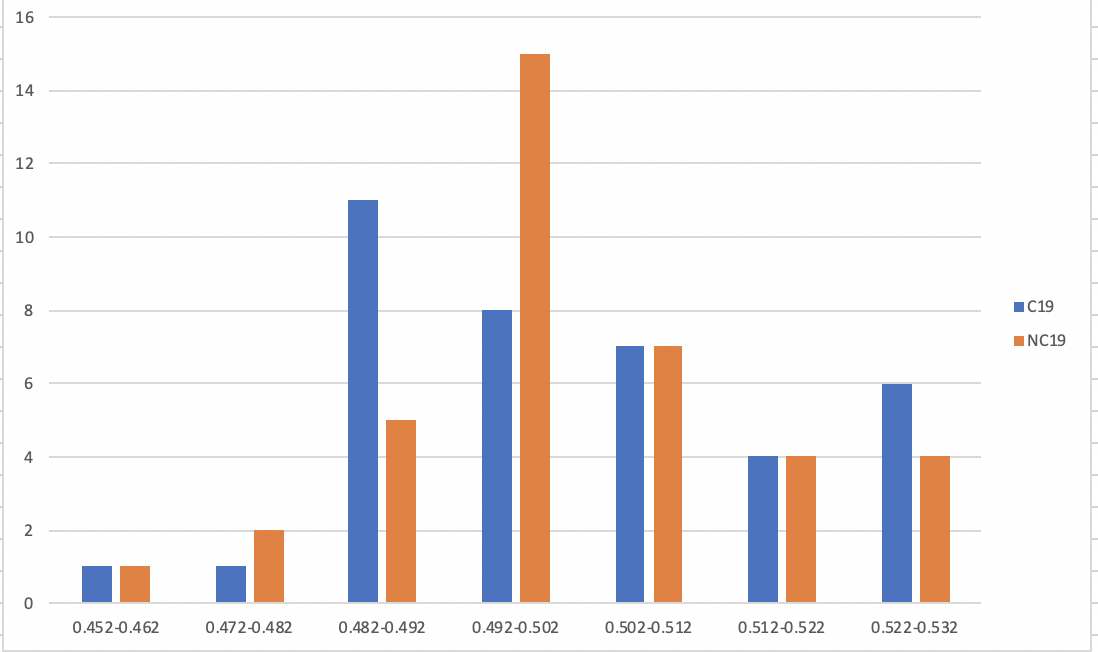
**

**Figure S1** Distributions of propensity scores for 38 COVID19 and 38 Non-COVID19 patients considering their age, gender, and length of stay.
