# Supplementary material for: Collaboration Structures in COVID-19 Critical Care: Retrospective Network Analysis Study
Source: JMIR Hum Factors. 2021 Mar 8;8(1):e25724. doi: 10.2196/25724 (PMC7942392; doi:10.2196/25724)
Supplement: Multimedia Appendix 2 [file humanfactors_v8i1e25724_app2.docx]

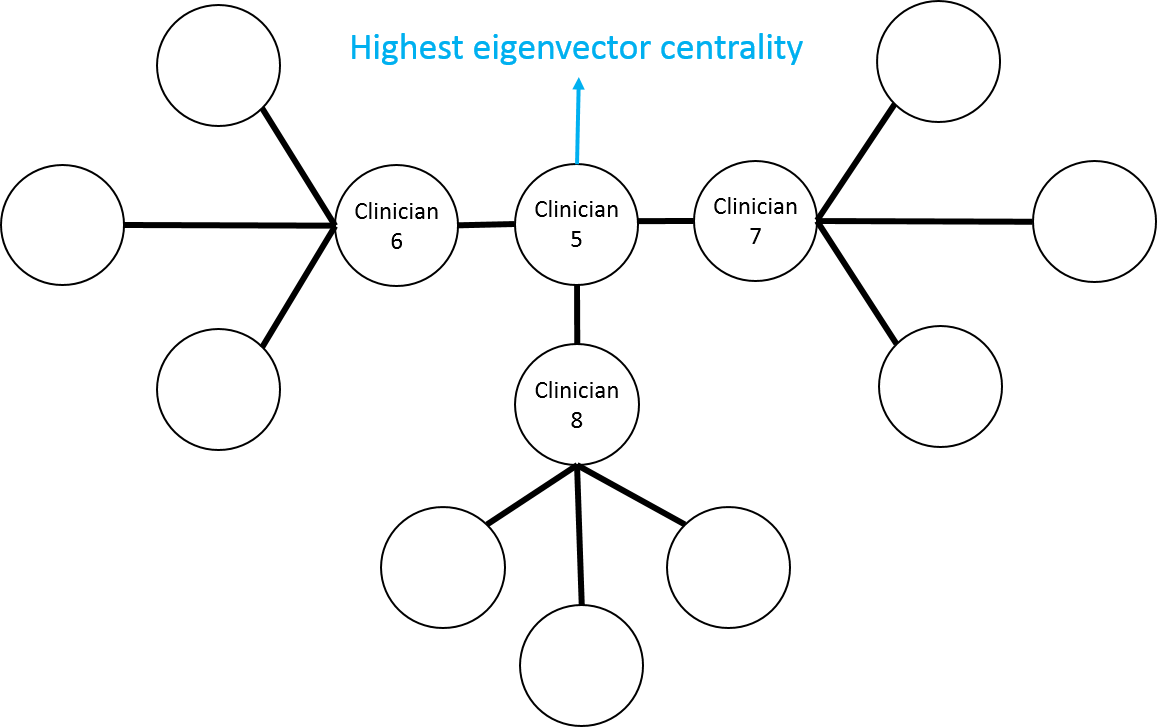


**Figure S2** An example of a clinician with highest eigencentrality.


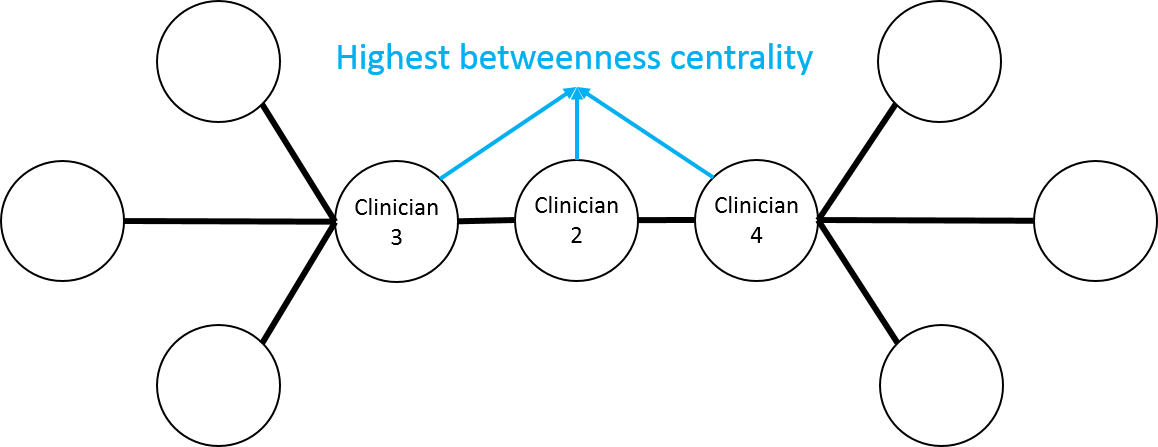


**Figure S3** An example of clinicians with highest betweenness centrality.
