# Supplementary material for: Collaboration Structures in COVID-19 Critical Care: Retrospective Network Analysis Study
Source: JMIR Hum Factors. 2021 Mar 8;8(1):e25724. doi: 10.2196/25724 (PMC7942392; doi:10.2196/25724)
Supplement: Multimedia Appendix 3 [file humanfactors_v8i1e25724_app3.docx]

There are 21 pairwise hypotheses tested, which makes the Bonferroni-corrected significance level as 0.0024 in both Tables A1 and A2.

**Table S1** Differences in the eigencentrality between COVID19(C19) and Non-COVID-19(NC19) networks**.** * indicates the difference is significant.

| **Expertise** | **Mean** | | **Median** | | **IQR** | | **Standard Error** | | **p-Value** |
| --- | --- | --- | --- | --- | --- | --- | --- | --- | --- |
|  | **C19** | **NC19** | **C19** | **NC19** | **C19** | **NC19** | **C19** | **NC19** |  |
| Med Center East+ : Registered Nurse | 0.06 | 0.02 | 0.06 | 0.02 | 0.06 | 0.02 | 0.01 | 0.00 | 5.53 × 10^-4^* |
| Internal Medicine : Physician | 0.18 | 0.05 | 0.12 | 0.03 | 0.25 | 0.06 | 0.04 | 0.01 | 1.24 × 10^-3^* |
| Medicine-Housestaff : Resident Physician | 0.04 | 0.11 | 0.02 | 0.08 | 0.03 | 0.11 | 0.01 | 0.02 | 2.26 × 10^-3^* |
| MICU : Registered Nurse | 0.18 | 0.09 | 0.11 | 0.07 | 0.24 | 0.08 | 0.03 | 0.01 | 1.74 × 10^-2^ |
| Hospital Nurse Practitioners : Nurse Practitioner | 0.49 | 0.12 | 0.65 | 0.08 | 0.81 | 0.11 | 0.09 | 0.03 | 2.91 × 10^-2^ |
| Nephrology : Physician | 0.16 | 0.23 | 0.05 | 0.22 | 0.11 | 0.22 | 0.07 | 0.05 | 3.47 × 10^-2^ |
| Nephrology : Fellow | 0.26 | 0.17 | 0.26 | 0.13 | 0.21 | 0.17 | 0.06 | 0.04 | 2.19 × 10^-1^ |
| Respiratory Care : Respiratory Therapist | 0.26 | 0.21 | 0.14 | 0.10 | 0.32 | 0.32 | 0.04 | 0.04 | 2.32 × 10^-1^ |
| Allergy/Pulmonary : Physician | 0.37 | 0.27 | 0.26 | 0.16 | 0.57 | 0.33 | 0.09 | 0.07 | 2.49 × 10^-1^ |
| Pharm Inpt Evening : Pharmacy Technician | 0.29 | 0.23 | 0.29 | 0.25 | 0.34 | 0.29 | 0.06 | 0.06 | 2.81 × 10^-1^ |
| Radiology : Physician | 0.14 | 0.11 | 0.05 | 0.05 | 0.09 | 0.15 | 0.06 | 0.04 | 4.03 × 10^-1^ |
| Emergency Medicine : Physician | 0.09 | 0.11 | 0.09 | 0.07 | 0.09 | 0.08 | 0.02 | 0.03 | 4.19 × 10^-1^ |
| +: Medical Center East is the physical building where COVID unit is created. Before the creation of the COVID unit, nurses in the building care for critically ill NC19 patients. | | | | | | | | | |

**Table S2** Differences in the betweenness between COVID19(C19) and Non-COVID19(NC19) networks. * indicates the difference is significant.

| **Expertise** | **Mean** | | **Median** | | **IQR** | | **Standard Error** | | **p-Value** |
| --- | --- | --- | --- | --- | --- | --- | --- | --- | --- |
|  | **C19** | **NC19** | **C19** | **NC19** | **C19** | **NC19** | **C19** | **NC19** |  |
| Hospital Nurse Practitioners : Nurse Practitioner | 0.22 | 0.02 | 0.10 | 1.61× 10^-3^ | 0.25 | 0.02 | 0.07 | 0.02 | 3.06 × 10^-4^* |
| Nephrology : Fellow | 0.01 | 2.49 × 10^-3^ | 3.52 × 10^-3^ | 2.30 × 10^-4^ | 0.01 | 3.58 × 10^-3^ | 4.59 × 10^-3^ | 1.57× 10^-3^ | 2.60 × 10^-2^ |
| Internal Medicine : Physician | 0.09 | 0.02 | 0.02 | 0.01 | 0.14 | 0.03 | 0.04 | 0.01 | 3.05 × 10^-2^ |
| Emergency Medicine : Physician | 0.01 | 0.03 | 8.11× 10^-4^ | 0.02 | 9.77× 10^-4^ | 0.06 | 0.01 | 0.01 | 3.09 × 10^-2^ |
| Medicine-Housestaff : Resident Physician | 3.47 × 10^-3^ | 0.03 | 7.34 × 10^-4^ | 0.01 | 4.90 × 10^-3^ | 0.02 | 1.94 × 10^-3^ | 0.01 | 3.21 × 10^-2^ |
| Respiratory Care : Respiratory Therapist | 0.02 | 0.05 | 3.50 × 10^-3^ | 0.01 | 0.01 | 0.06 | 0.01 | 0.02 | 7.43 × 10^-2^ |
| MICU : Registered Nurse | 0.01 | 0.01 | 1.81 × 10^-3^ | 5.09 × 10^-4^ | 0.02 | 2.88 × 10^-3^ | 3.06 × 10^-3^ | 1.71 × 10^-3^ | 1.23 × 10^-1^ |
| Pharm Inpt Evening : Pharmacy Technician | 0.01 | 0.03 | 1.24 × 10^-3^ | 0.01 | 0.02 | 0.04 | 0.01 | 0.01 | 2.05 × 10^-1^ |
| Allergy/Pulmonary : Physician | 0.11 | 0.17 | 0.07 | 0.08 | 0.13 | 0.14 | 0.03 | 0.07 | 3.76 × 10^-1^ |
