# Supplementary material for: Collaboration Structures in COVID-19 Critical Care: Retrospective Network Analysis Study
Source: JMIR Hum Factors. 2021 Mar 8;8(1):e25724. doi: 10.2196/25724 (PMC7942392; doi:10.2196/25724)
Supplement: Multimedia Appendix 4 [file humanfactors_v8i1e25724_app4.docx]

**Table S3** Differences in the total number of healthcare workers, expertise, and actions performed to EHRs of a patient with and without COVID19 during their hospital stays. Differences in the number of healthcare workers, expertise, and actions performed to EHRs of a patient with and without COVID19 on a daily basis. Six paiwise hypotheses were tested, which makes the Bonferroni-corrected significance level as 8.33 × 10^-3^. None of the hypotheses were confirmed.

|  | **COVID19 structure** | **Non-COVID19 structure** |
| --- | --- | --- |
| **Patient staffing intensity across the entire hospital stay** | | |
| **Number of actions** | | |
| Mean | 967.66 | 936.24 |
| Median | 742.00 | 774.00 |
| IQR | 810.00 | 898.00 |
| Standard Error | 167.33 | 114.22 |
| p Value | 0.32 | |
|  | | |
| **Number of distinct healthcare workers** | | |
| Mean | 79.50 | 88.18 |
| Median | 72.00 | 79.50 |
| IQR | 68.00 | 69.50 |
| Standard Error | 7.87 | 7.84 |
| p Value | 0.19 | |
|  | | |
| **Number of distinct expertise** | | |
| Mean | 29.16 | 33.95 |
| Median | 30.50 | 31.00 |
| IQR | 15.50 | 22.50 |
| Standard Error | 1.71 | 2.25 |
| p Value | 0.08 | |
| **Daily basis** | | |
| **Number of actions per day** | | |
| Mean | 57.78 | 59.43 |
| Median | 55.46 | 60.03 |
| IQR | 29.43 | 18.62 |
| Standard Error | 3.90 | 2.62 |
| p Value | 0.19 | |
|  | | |
| **Number of distinct healthcare workers per day** | | |
| Mean | 5.59 | 6.28 |
| Median | 5.36 | 6.19 |
| IQR | 2.28 | 2.43 |
| Standard Error | 0.27 | 0.28 |
| p Value | 4.98 × 10^-2^ | |
|  | | |
| **Number of distinct expertise per day** | | |
| Mean | 2.44 | 2.74 |
| Median | 2.17 | 2.53 |
| IQR | 1.19 | 1.56 |
| Standard Error | 0.18 | 0.21 |
| p Value | 0.15 | |
